# Effects of prenatal low protein and postnatal high fat diets on visceral adipose tissue macrophage phenotypes and IL-6 expression in Sprague Dawley rat offspring

**DOI:** 10.1371/journal.pone.0169581

**Published:** 2017-01-31

**Authors:** Linglin Xie, Ke Zhang, Dane Rasmussen, Junpeng Wang, Dayong Wu, James N. Roemmich, Amy Bundy, W. Thomas Johnson, Kate Claycombe

**Affiliations:** 1 Department of Nutrition and Food Sciences, Texas A&M University, College Station, Texas, United States of America; 2 Department of Basic Sciences, School of Medicine and Health Sciences, University of North Dakota, Grand Forks, North Dakota, United States of America; 3 Department of Pathology, School of Medicine and Health Sciences, University of North Dakota, Grand Forks, North Dakota, United States of America; 4 ND INBRE Bioinformatics Core, University of North Dakota, Grand Forks, North Dakota, United States of America; 5 Jean Mayer USDA Human Nutrition Research Center on Aging at Tufts University, Boston, Massachusetts, United States of America; 6 USDA Agricultural Research Service, Grand Forks Human Nutrition Research Center, Grand Forks, North Dakota, United States of America; State University of Rio de Janeiro, BRAZIL

## Abstract

Adipose tissue macrophages (ATM) are implicated in adipose tissue inflammation and obesity-related insulin resistance. Maternal low protein models result in fetal programming of obesity. The study aims to answer whether maternal undernutrition by protein restriction affects the ATM M1 or M2 phenotype under postnatal high fat diet in F1 offspring. Using a rat model of prenatal low protein (LP, 8% protein) diet followed by a postnatal high fat energy diet (HE, 45% fat) or low fat normal energy diet (NE, 10% fat) for 12 weeks, we investigated the effects of these diets on adiposity, programming of the offspring ATM phenotype, and the associated inflammatory response in adipose tissue. Fat mass in newborn and 12-week old LP fed offspring was lower than that of normal protein (20%; NP) fed offspring; however, the adipose tissue growth rate was higher compared to the NP fed offspring. While LP did not affect the number of CD68^+^ or CD206^+^ cells in adipose tissue of NE offspring, it attenuated the number of these cells in offspring fed HE. In offspring fed HE, LP offspring had a lower percentage of CD11c^+^CD206^+^ ATMs, whose abundancy was correlated with the size of the adipocytes. Noteworthy, similar to HE treatment, LP increased gene expression of *IL-6* within ATMs. Two-way ANOVA showed an interaction of prenatal LP and postnatal HE on *IL-6* and *IL-1β* transcription. Overall, both LP and HE diets impact ATM phenotype by affecting the ratio of CD11c^+^CD206^+^ ATMs and the expression of *IL-6*.

## Introduction

Epidemiologic studies have shown that low birth weight (LBW) is associated with increased incidence of obesity, coronary heart disease, type 2 diabetes and metabolic syndrome [1–5]. A well-established cause of LBW is maternal under nutrition–induced intrauterine growth restriction (IUGR) [6, 7]. As proposed in the thrifty phenotype hypothesis [8], maternal under-nutrition slows fetal growth. This enhances the fetus’ ability to survive by reserving nutrients for developing critical organs, such as brain, kidney and heart, at the expense of adipose, muscular and skeletal tissue development. However, when these offspring are exposed to over-nutrition, the greater ability to efficiently store nutrients results in obesity in later life [9–11]. In agreement with this hypothesis, IUGR results in subsequent postnatal catch-up growth and development of obesity [12, 13]. Maternal low protein models of fetal programming have been widely used to investigate the mechanisms linking maternal nutrition with F1 obesity [14–16]. A common trait in the F1 offspring is an age-related loss of glucose tolerance and development of insulin resistance [17–20]. In order to understand the underlying mechanisms for the glucose intolerance associated with IUGR, studies have focused on the alterations in insulin secretion and action that occur during catch-up growth [21].

The obesity epidemic has resulted in an explosion of obesity-related health problems, including insulin resistance and type II diabetes. The chronic low-grade inflammation that occurs within the adipose tissue of obese subjects contributes to pathogenesis of insulin resistance [22]. Macrophages are the major adipose tissue-resident immune cell types involved in the development of chronic inflammation [22–24]. The adipose tissue of obese mice has a 2- to 5-fold increase in macrophage infiltration, along with higher systemic levels of macrophage-secreted inflammatory cytokines [22].

Macrophages show heterogeneity in their function depending on the resident microenvironment. Classically activated or M1 macrophages (CD11c^+^) produce pro-inflammatory cytokines (e.g. TNF-α, IL-1β and IL-6) and are the predominant type of adipose tissue macrophages (ATMs) in dietary-induced obese (DIO) humans and animals [25, 26]. M1 macrophages are sub-divided into M1a and M1b types based on the absence or presence of CD206, respectively [27]. Alternatively activated or M2 macrophages (CD11c^-^CD206^+^) secret anti-inflammatory cytokines (e.g. IL-4, IL-10 and IL-1 receptor antagonist) and are a dominant population of ATM in lean mice [26, 28, 29]. In contrast to the progress in defining the role of ATM in the pathogenesis of obesity-related insulin resistance, little is known of how maternal undernutrition influences ATM phenotypes of offspring consuming normal or high energy diets.

Using a rat model of a prenatal low protein (LP, 8% protein) diet followed by a normal or a postnatal high fat energy diet (HE, 45% fat) for 12 weeks, effects of these diets on programming of the offspring ATM phenotype were investigated in the current study. We found that maternal LP did not affect the number of CD68+ or CD206+ cells in adipose tissue of NE offspring, but increased the gene expression of IL-6 and IL-1β. However, maternal LP diet interact with postnatal HE diet interacts on the catch up growth and the enlargement of the offspring adipocyte, which further correlates with the ATM phenotype.

## Materials and methods

### Study design

Two month old obese-prone Sprague-Dawley male and female rats were purchased from Charles River (Wilmington, MA) and maintained on a chow diet for 2 weeks prior to the start of experiment diets. Twelve female Sprague Dawley rats were placed on either the control (NP, 20% protein, *n* = 12 litters) or low-protein (LP, 8% protein, *n* = 12 litters) diet 2 days after conception and remained on this diet throughout gestation and lactation. The females were mated for 2 days. Pregnancy was confirmed when plugs were found. Pregnant females were then placed in a single cage and weighed weekly for 3 week gestational period. At birth, litter size and birth weight were recorded and offspring (F1) were randomly culled to 8 pups (4 male, 4 female). We used 4 male pups from each dam for further experiments. At weaning, one-half of the male offspring born to dams fed LP were placed on a diet containing an energy density of 3.84 kcal/g (10% fat energy, 70% carbohydrate energy, and 20% protein energy; hence referred to as the NE—normal energy diet), the other half were given a diet containing 4.73 kcal/g (45% fat energy, 20% carbohydrate energy, and 20% protein energy; hence referred to as the HE—high energy diet) (Table 1). Because rats fed HE ate less, these diets had higher concentrations of vitamins, minerals, and protein such that these nutrients in the HE diet were equivalent to the NE diet on a per kcal basis (Table 2). Offspring were divided into four groups: NP+NE, LP+NE, NP+HE, and LP+HE. Offspring were maintained on these diets for 12 weeks. The food efficiency was determined by dividing body weight gain of each experimental mice by the total energy intake.

**Table 1:** Prenatal low and normal protein diet composition.

	Normal Protein (NP)		Low Protein (LP)	
Ingredient	g	kcal	g	kcal
Casein	200	800	80	320
L-cysteine	3	12	3	12
Corn Starch	310	1240	390.82	1563.3
Maltodextrin	35	140	35	140
Sucrose	297.5	1190	337.91	1351.6
Cellulose	50		50	
Soybean oil	25	225	25	225
Lard	20	180	20	180
CaCO_3_	12.5		7.27	
CaHPO_4_			4	
Mineral mix	35		35	
Vitamin mix	10	40	10	40
Choline bitartrate	2		2	
Total	1000	3827	1000	3827
	wt%	kcal%	wt%	kcal%
Protein	20.3	21.22	8.3	8.66
Carbohydrate	67.24	67.26	79.36	79.83
Fat	4.5	10.58	4.5	10.57

**Table 2:** Postnatal normal and high fat diet composition.

	Normal Protein (NP)		Low Protein (LP)	
Ingredient	g	kcal	g	kcal
Casein	200	800	80	320
L-cysteine	3	12	3	12
Corn Starch	315	1260		
Maltodextrin	35	140	125	500
Sucrose	350	1400	215	860
Cellulose	50		50	
Soybean oil	25	225	25	225
Lard	20	180	20	180
CaCO_3_	12.5		12.5	
Mineral mix	35		35	
Vitamin mix	10	40	10	40
Choline bitartrate	2		2	
Total	1057.5	4057	857.5	4057
	3.84 kcal/g		4.73 kcal/g	
	wt%	kcal%	wt%	kcal%
Protein	19.20	20.01	23.67	20.01
Carbohydrate	66.19	69.12	43.13	33.62
Fat	4.26	9.98	23.91	45.48

All animals were caged in a controlled environment with a 12-hour light, 12-hour dark cycle and received pathogen-free water. At the end of 12-week postnatal diets, rats were injected with xylazine (Rompon, Moboay Inc., Shawnee, KS) and ketamine (Ketaset, Aveco Inc., Fort Dodge, IA) and sacrificed by exsanguinations according to the USDA ARS animal care and use committee guidelines.

The animal use and care protocol was approved by USDA Agricultural Research Service Animal Care and Use Committee (Permit Number: Claycombe-Prog04). Rats were injected with xylazine (Rompon, Moboay) and ketamine (Ketaset, Aveco) and killed by exsanguination, and all efforts were made to minimize suffering.

### Antibodies

Mouse anti-rat CD11b/c PerCP-eFluor^®^ 710 (Cat#46–0110) and its isotype control (Cat#46–4724) for flow cytometry were purchased from eBioscience (San Diego, CA). Anti-Mouse Ig APC (Cat# 550826) for flow cytometry was purchased from BD bioscience (San Jose, CA) and Donkey Anti-Rabbit IgG PE (Cat#12-4739-81) was from eBioscience (San Diego, CA). Anti-rat CD68 FITC (Cat#SM1550F) for flow cytometry was from Acris-antibodies (San Diego, CA). Rabbit anti-rat CD206 (Mannose Receptor antibody) (Cat#ab64693), mouse anti-rat CD11c (Cat#ab11029) and mouse anti-rat CD68 (Cat#ab53444) for immunohistochemical staining were from Abcam (Cambridge, MA).

### EchoMRI measurements of body composition

Whole body composition (fat mass, lean mass, and total body water) was determined biweekly without any sedation using nuclear magnetic resonance technology with EchoMRI700^™^ instrument (Echo Medical Systems, Houston, TX) during the 12 week postnatal diet period.

### Stromal Vascular Cell (SVC) isolation and FACS analysis

Epididymal fat pads were weighed, rinsed 3 times in phosphate-buffered saline (PBS), and the SVCs were isolated as described previously [30]. SVCs were immunostained with primary antibodies for 30 min at 4°C followed by incubation with fluorescent-bounded secondary antibody for another 30 min at 4°C in dark. SVCs were analyzed using an Accuri C6 flow cytometer (BD Accuri Cytometers, Ann Arbor, MI), and data were analyzed with FlowJo 7.6 software (Treestar Inc., Ashland, OR).

### Adipose tissue analysis

Experimental offspring mice were euthanized by CO_2_ inhalation. Visceral white adipose tissue was immediately collected and fixed in 10% formalin/PBS. The tissue was embedded in paraffin blocks after processing and was cut into 5-μm-thick slices using a TC-2 tissue sectioner (Sorvall Instruments). Tissue slices were mounted onto positive pre-charged glass slides to ensure optimal adhesion. CD68 (Acris-antibodies, San Diego, CA), CD206 (Abcam, Cambridge, MA) and CD11c (Abcam, Cambridge, MA) were immunostained using a VECTASTAIN Elite Avidin/Biotin-Complex (ABC) kit for mouse IgG or a VECTASTAIN Elite ABC kit for rabbit IgG (Vector Laboratory). Staining of the tissue was visualized under an Olympus BH-2 microscope and pictures were taken using a Leica M165FC camera and Leica Application Suit V3 was used for picture processing. The number of positive cells per 1000 adipocytes in each staining section was blindly counted. The average of the five counts was used for data analysis. Adipocyte diameter was measured using Image J software. Two hundred cells were randomly counted in each sample. The adipocytes of five animals from each group were counted. An average diameter was recorded for each animal.

### Isolation of CD68^+^ macrophages from SVCs

SVCs were treated with 1% Tween-20 PBS for 30 minutes at room temperature. Then SVCs were incubated with anti-rat CD68 FITC (Acris-antibodies, San Diego, CA) for 30 minutes at 4°C in dark and washed with Magnetic Cell Separation (MACS) buffer (Miltenyi Biotec Inc., Auburn, CA). SVCs were then incubated with anti-FITC MicroBeads (Miltenyi Biotec Inc., Auburn, CA) for 15 minutes at 4°C in dark and washed twice with MACS buffer. The pellets were re-suspended in 500 μl MACS buffer and flow through MS columns (Miltenyi Biotec Inc., Auburn, CA) according to the manufacturer’s manual. The FITC MicroBeads bound CD68^+^ cells were collected for RNA extraction.

### Real-time PCR

Total RNA of CD68^+^ cells from SVCs was extracted using Invitrogen Trizol reagent. cDNA was synthesized using Qiagen RT^2^ First Strand Kit (Qiagen, Valencia, CA). Primers were designed as listed in Table 3. Real time PCR was performed using an ABI Prism 7500 PCR system (Applied Biosystems, Foster City, CA). The ΔCT values were used for statistical analysis for real time-PCR experiments. The standard deviation of the fold change in gene expression for real time-PCR data was derived by the delta method [31].

**Table 3:** Primers used for RT-PCR experiment.

Gene	forward primer	reverse primer
*IL-6*	5’-ggtttgccgagtagacctca-3’	5’-gtggctaaggaccaagacca-3’
IL-1β	5’- aaagaaggtgcttgggtcct-3’	5’- caggaaggcagtgtcactca-3’
MCP-1	5’- ccgactcattgggatcatct-3’	5’- tagcatccacgtgctgtctc-3’
*Arg1*	5’- gacatccacaaaggccagat-3’	5’- tatcggagcgcctttctcta-3’
*IL-10*	5’-tgggaagtgggtgcagttat-3’	5’- gctcagcactgctatgttgc-3’

### Statistical analysis

The normality of data was ensured using Shapiro-Wilk test. Differences between the groups were analyzed using Fishers’ Least Significant Difference (LSD) test such that the multiple comparisons between groups were taken into account. Fisher’s LSD test was performed by first carrying out one-way analysis of variance (ANOVA) for all four treatment groups and then conducting two-group pairwise t-tests only for outcomes showing statistical significance in the ANOVA tests. Because two factors, maternal (LP vs NP) and postnatal (HE vs NE), were involved in the experimental design, two-way ANOVA was used to assess the main effects and the interaction between the two factors. The correlation between the ratio of M1b cells to M1 cells and the sizes of adipose cells were analyzed using ordinary least squares regression analysis. The M1b ratio data were logarithm transformed prior to analysis because Breusch-Pagan test found that the original ratios did not fit a linear model. All analyses were carried out using SAS^®^ JMP software and R statistical programming language. The significance level of statistical test was defined as P<0.05. Data in graphs are presented as mean ± SEM and sample size n. Bars bearing different letters indicated significant difference between the bars (P<0.05).

## Results

### Prenatal LP offspring had lower adipose tissue mass and higher adipose tissue growth rate compared to NP offspring

**Fig 1:**
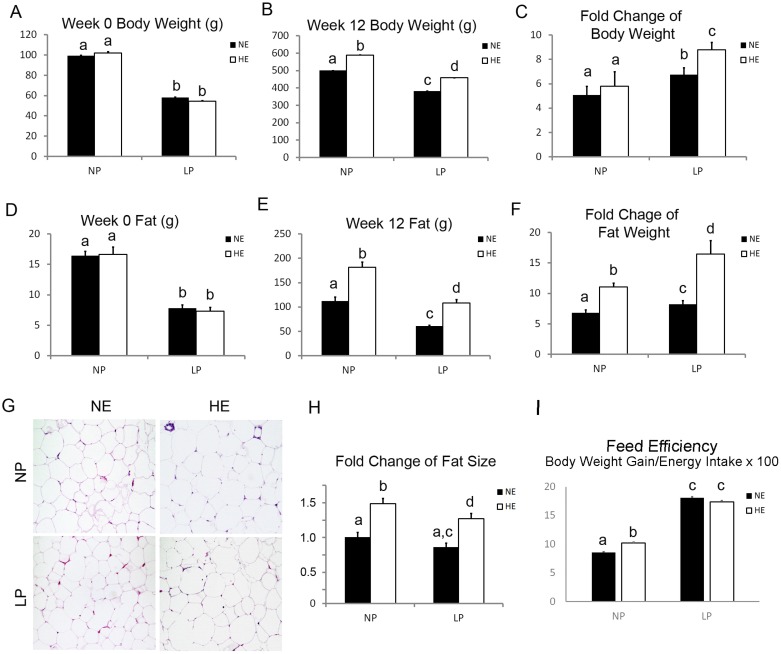
Prenatal LP offspring had reduced adipose tissue mass but significantly increased adipose tissue gain. A and B). Body weight of the experimental rats was measured at week 0 (weaning weight) and week 12. Data are presented as mean ± SEM, n = 9–12. C). Body weight change was calculated by dividing the body weight (g) and then subtracted one at week 12 by the weaning body weight (g). Data are presented as mean ± SEM, n = 9–12. D-E) Body fat mass was measure by EchoMRI at week 0 (weaning weight) and week 12. Data are presented as mean ± SEM, n = 9–12. F) Body fat change was calculated by dividing the fat weight (g) at week 12 by the weaning fat weight (g). Data are presented as mean ± SEM, n = 9–12. Bars bearing different letters significantly differ at P<0.05. G and H) Visceral fat tissue was collected for tissue slide sectioning and H&E staining. Eight hundred adipocytes from each rat sectioning were randomly picked up and the size was measured by ImageJ. Five rats were counted in each group. Size of the adipocytes was normalized to that of the NP+NE rats. Data are presented as mean ± SEM, n = 5. Bars bearing different letters significantly differ at P<0.05. I). The feed efficiency was calculated by “body weight gain/energy intake x100”. Data are presented as mean ± SEM, n = 5. Bars bearing different letters significantly differ at P<0.05.

LP offspring had lower body and adipose tissue weights at weaning compared to the NP offspring (Fig 1A and 1D). While these prenatal LP-induced differences remained significant at postnatal week 12, offspring fed the postnatal HE diets had greater body weights and body fat mass than the NE rats, regardless of prenatal diet (Fig 1B and 1E). However, when calculated as fold change, LP (vs. NP) increased body weight gain independent of postnatal diets, while HE diets only increased body weight gain in LP offspring, but not in NP offspring (Fig 1C). The prenatal LP and postnatal HE synergistically increased the weight gain and fat gain of offspring, with LP+HE rats having the largest catch-up weight gain and fat gain compared to NP+HE rats (Fig 1C and 1F).

Offspring fed postnatal HE had larger visceral adipocytes compared to offspring fed NE. However, the prenatal LP diet diminished this effect of HE because LP+HE rats had smaller adipocytes compared to NP+HE rats (Fig 1G and 1H). Since feed efficiency (body weight gain/ energy intake) is an important contributing factor in adipose tissue weight gain, we therefore calculated the feed efficiency of the offspring. The results showed that feed efficiency were significantly increased in LP offspring, but not in the HE offspring, although feed efficiency in NP+HE offspring was slightly higher than NE+NP offspring. Clearly, this result does not explain the adipose tissue weight gain (Fig 1I).

### Prenatal LP prevented CD68^+^ and CD206^+^ cell infiltration in visceral adipose tissue of DIO rats

**Fig 2:**
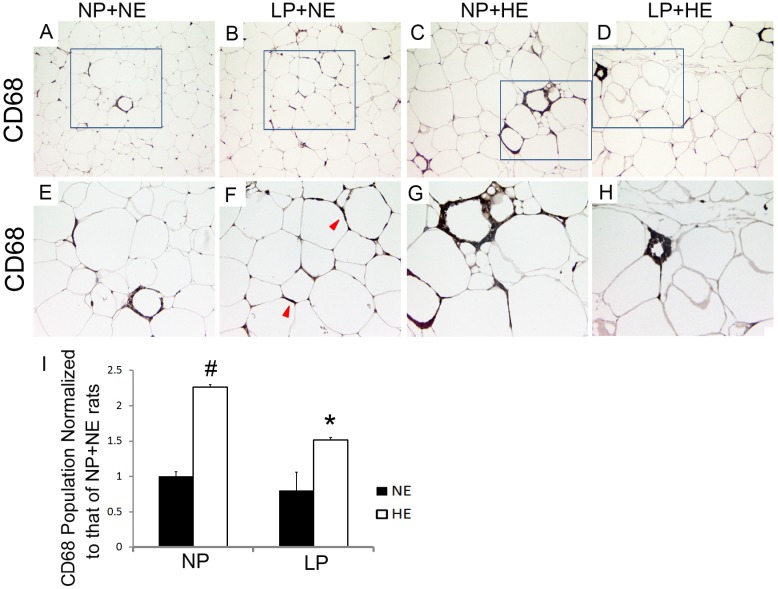
Prenatal LP prevented CD68^+^ ATM infiltration triggered by HE diets. A-I) ATMs were stained with anti-CD68 antibody. The number of ATMs was counted per 1,000 adipocytes area. Three randomly selected areas were counted for each rat. Average number of the ATMs was used for further statistical analysis. Number of CD68 positive cells in each group is normalized to the number of CD68^+^ macrophages in NP+NE group. Data are presented as mean ± SEM, n = 6–9.”#” P<0.05 compared to NE; “*” P<0.05 compared to NP. E, F, G, and H are enlargements of the framed fields of A, B, C, and D, respectively. Magnificence of A-D: 200X; E-H 400X.

We have summarized the macrophage markers used in our study for M1, M2 and subtypes of M1 macrophages in Table 4. The number of CD68^+^ cells in each group was normalized to the number of CD68^+^ macrophages in the NP+NE group. More ATM were observed in HE offspring compared to NE offspring regardless of prenatal diet (HE vs. NE on NP diet: 2.44±0.05 vs. 1.00±0.11, P<0.01; HE vs. NE on LP diet: 0.89±0.27 vs. 1.53±0.03, P<0.05). The prenatal LP diet decreased the number of CD68^+^ ATM only in offspring fed HE diets (Fig 2A–2I) (LP vs. NP on HE diet: 2.44±0.05 vs. 1.53±0.03, P<0.01).

**Table 4:** Identification markers used in our study to classify macrophage phenotypes.

Classification	Surface marker		
M1	CD11b/c^+^CD11c^+^	M1a	CD11b/c^+^CD11c^+^ CD206^-^
		M1b	CD11b/c^+^CD11c^+^ CD206^+^
M2	CD11b/c^+^ CD11c^-^CD206^+^		

**Fig 3:**
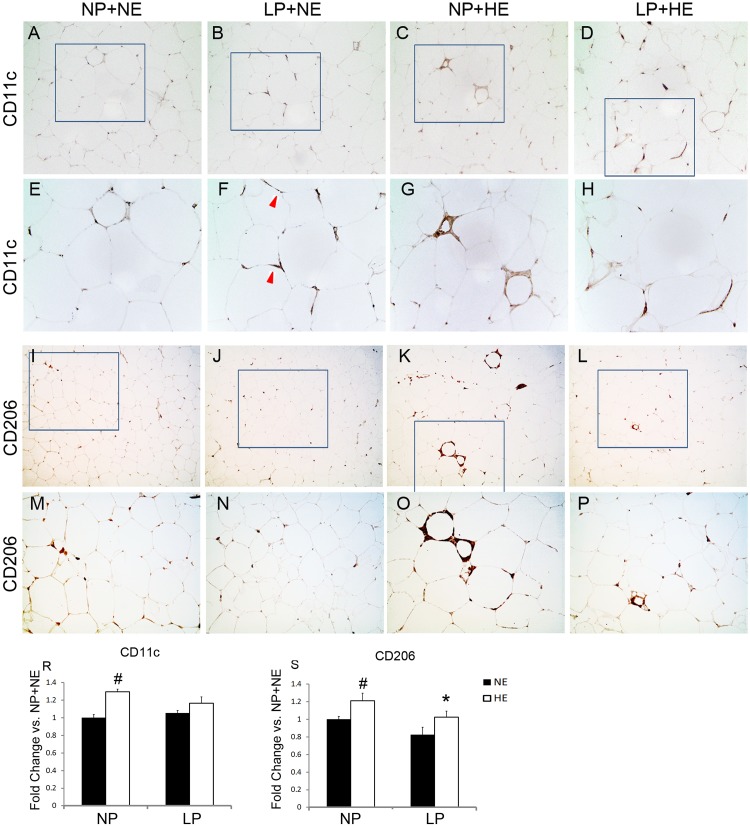
HE diets enhanced number of CD11c^+^ or CD206^+^ cells in visceral adipose tissue, while LP inhibited the increase of CD11c^+^ or CD206^+^ cells due to HE diets. A-H and R). Visceral adipose tissue was stained with anti-CD11c. Number of CD11c^+^ cells was counted per 1,000 adipocytes area. Three randomly selected areas were counted for each rat. Number of CD11c positive cells in each group is normalized to the number of CD11c^+^ macrophages in NP+NE group. Average number of the CD11c^+^ was used for further statistical analysis. Data are presented as mean ± SEM, n = 5.”#” P<0.05 compared to NE; “*” P<0.05 compared to NP. E, F, G, and H are enlargements of the framed fields of A, B, C, and D, respectively. I-P and S). Visceral adipose tissue was stained with anti-CD206. Number of CD206^+^ cells was counted per 1,000 adipocytes area. Three randomly selected areas were counted for each rat. Number of CD206 positive cells in each group is normalized to the number of CD206^+^ macrophages in NP+NE group. Average number of the CD206^+^ was used for further statistical analysis. Data are presented as mean ± SEM, n = 9–12. M, N, O, and P are enlargements of the framed fields of I, J, K, and L, respectively. Magnificence of A-D and I-L: 200X; E-H and M-P 400X.

CD11c^+^ cells (Fig 3A–3H) and CD68^+^ cells (Fig 2A–2H) were highly recruited to the crown like structure (CLS), but much more scattered in non-CLS regions of rats from HE groups. Interestingly, CD68^+^ or CD11c^+^ cells aggregate more around adipocytes in LP+NE rats than in NP+NE rats. CD11c^+^ cells were much more frequent in NP+HE rats compared to NP+NE rats (HE vs. NE on NP diet: 1.30±0.02 vs. 1.00±0.03, P<0.01). However, the difference in CD11c^+^ cell populations between LP+HE and LP+NE rats was not significant (Fig 3R). Overall, LP did not change the number of CD11c^+^ cells in adipose tissue. There were 21.3% more CD206^+^ cells in NP offspring with HE diets compared to those with NE diets (Fig 3S) (HE vs. NE on NP diet: 1.21±0.12 vs. 1.00±0.03, P<0.05). Whereas HE increased CD206^+^ cells in NP offspring, LP decreased CD206^+^ population in rats fed the HE diet, but not in rats fed the NE diet (Fig 3S) (LP vs. NP on HE diet: 1.21±0.12 vs. 1.03±0.04, P<0.05).

### HE diet increased the percentage of M1b ATM, while LP inhibited ATM phenotype switch to M1b in rats fed with HE diets

**Fig 4:**
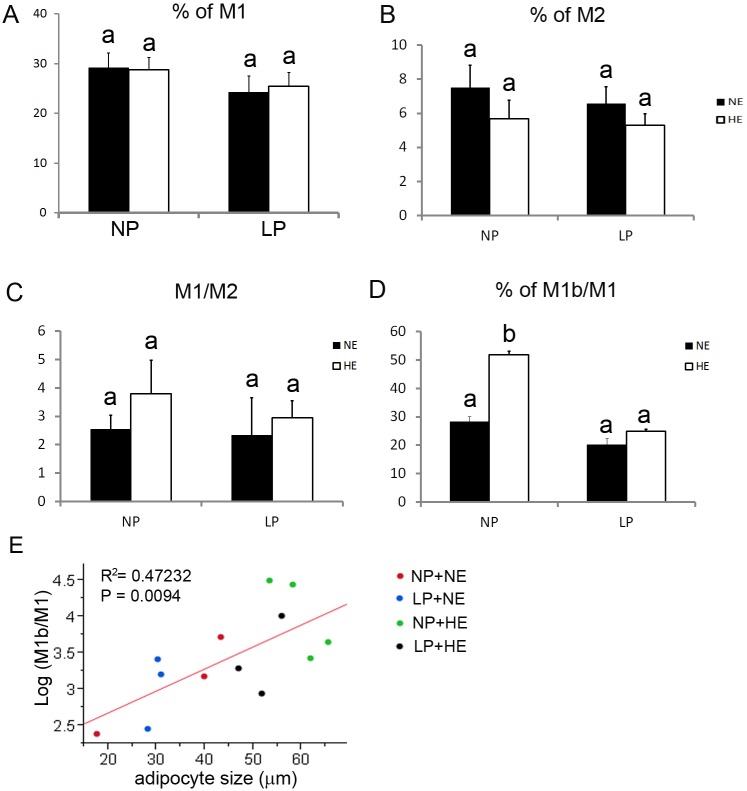
LP and HE impacts on ATMs plasticity. Isolated adipose tissue SVCs were stained with anti-CD11b/c PerCP-eFluor^®^ 710, anti-CD11c PE, anti-CD206 FITC for flow cytometry analysis. The M1% (A) and the M2% (B) were measured within the CD11b/c^+^, marker for monocytes/macrophages population. Data are presented as mean ± SEM, n = 9–12. Bars bearing different letters significantly differ at P<0.05.

The percentage of CD11c^+^ cells among CD11b/c^+^ population was not changed by either HE or LP diets (Fig 4A). There was no change in percentage of CD11c^-^CD206^+^ among the CD11b/c^+^ population due to LP or HE diets (Fig 4B). The M1/M2 ratio also was not changed among all four groups (Fig 4C). However, the percentage of CD11c^+^CD206^+^ in total CD11c^+^ (M1b/M1) was enhanced by HE in NP fed rats (Fig 4D) (HE vs NE on NP diet: 51.1%±0.9% vs. 28.8%±1.7%, P<0.05). However, a prenatal LP diet blocked the increase in M1b induced by HE diets (Fig 4D, HE+NP vs. HE+LP) (LP vs NP on HE diet: 25.4%±0.7% vs. 51.1%±0.9%, P<0.01). The percentage of M1b in M1 population was associated with the size of adipocytes (Fig 4E) (R^2^ = 0.47232, P = 0.0094).

### Prenatal LP and postnatal HE interacted on altering IL-6 and IL-1β transcription in ATMs

**Fig 5:**
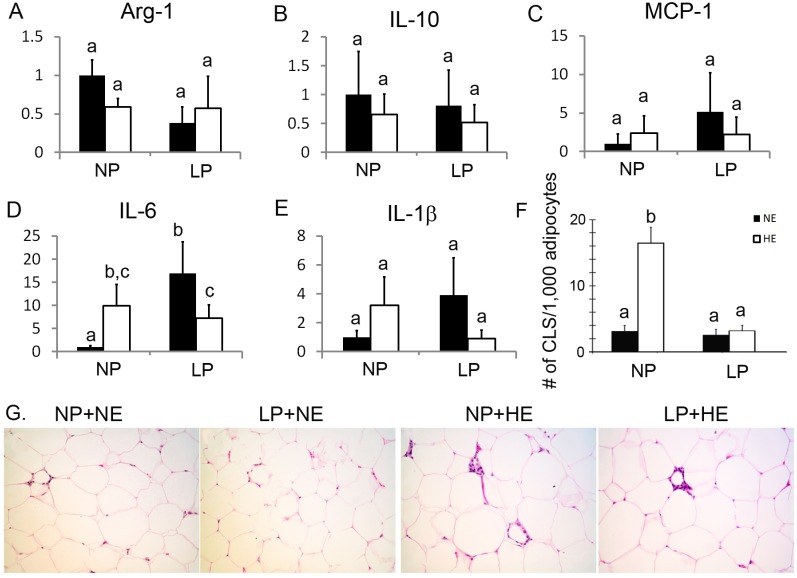
LP increased *IL-6* and *IL-1β* expression in isolated ATMs. A-E) Expression of *Arg-1*, *IL-10*, *MCP-1*, *IL-6* and *IL-1β* were detected by real-time PCR analysis. Data are presented as mean ± SEM, n = 3. Bars bearing different letters significantly differ at P<0.05. F and G) H&E staining on visceral adipose tissue. The number of CLS was counted per 1,000 adipocytes area. Five randomly selected areas were counted for each rat. Average number of the CLS was used for further statistical analysis. Data are presented as mean ± SEM, n = 9–12. Bars bearing different letters significantly differ at P<0.05. Magnificence of panel G is 400X.

There were no changes in the expression of M2 macrophage marker genes *IL-10* and *Arg-1* or in gene expression of *MCP-1* in any group, (Fig 5A–5C). Expression of *IL-6* was increased in NP+HE rats compared to NP+NE rats (Fig 5D) (HE vs. NE on NP diet: 10.0±3.8 vs. 1.0±0.04, P<0.05). *IL-6* expression was higher in LP compared to NP offspring (Fig 5D) (LP vs. NP on NE diet: 16.8±7.2 vs. 1.0±0.04, P<0.05). Surprisingly, LP combined with HE did not enhance *IL-6* expression in a synergic manner. Instead, LP partially prevented the increase in *IL-6* expression in HE rats, although *IL-6* expression still remained higher in LP+HE rats than NP+NE rats (Fig 5D) (LP vs. NP on HE diet: 16.8±7.2 vs. 7.1±2.4, P<0.05). We did not observe any transcriptional difference of *IL-1β* among all four groups (Fig 5E). However, two-way ANOVA analysis indicated that prenatal LP is a contributing factor that significantly altered the expression of *IL-1β* and *IL-6* (Table 5). In addition, the interaction evaluation by two-way ANOVA demonstrated antagonistic effect of LP and HE in regulating transcription of both *IL-6* and *IL-1β* (Table 5).

**Table 5:** P value of Two-way ANOVA for LP and HE on ATM phenotype^a^.

	HE (P value)	LP (P value)	LP × HE (P value)
*IL-6*	0.0003**	< .0001**	< .0001**
*IL-1β*	0.0048**	0.0016**	0.0043**
*Ccl2*	0.3276	0.0825	0.1816
*IL-10*	0.4826	0.7238	0.9701
*Arg-1*	0.4897	0.2649	0.3888
Number of CD68^+^	< .0001**	0.4229	0.104
Number of CD11c^+^	0.0008**	0.4644	0.0852
Number of CD206^+^	0.0364*	0.0904	0.5771
Number of CLS	< .0001**	0.688	< .0001**

Note:

^a^ The significant impacts of the two factors: postnatal HE diet and the maternal LP diet, and the interactions between these two factors on ATM phenotype and cytokine gene expression were analyzed by Two-way ANOVA.

** P<0.01,

* P<0.05

Haematoxylin Eosin (H&E) staining of visceral adipose tissue showed that CLS was more frequent in HE rats compared to NE rats (HE vs NE on NP diet, 15±2 vs. 3±1, P<0.05). While LP did not change CLS in NE rats, it decreased the number of CLS in HE rats (Fig 5F–5J). There was an interaction of LP and HE in the formation of CLS (Table 5, P < .0001).

## Discussion

Adipose tissue inflammation, contributed by macrophage infiltration into the adipose tissue, is an important step in the pathogenesis of obesity-related complications. These ATM are phenotypically heterogeneous and present different plasticity in lean versus obese subjects [26]. ATM characteristics have been well described in high-fat diet-induced obesity or genetic-related obesity [22, 26, 32, 33]. Previous study has reported that a low protein diet during pregnancy affects lymphocyte and complementary systems [34, 35]. However, there is limited information whether prenatal programming, affected by maternal protein restriction, changes ATM heterogeneity. It is also unknown whether maternal low protein diet and postnatal high fat diet interact to influence ATM phenotype. We reported that LP had no effect on the number of CD68+ and CD206+ in adipose tissue of NE rats, but reduced the increase of these cell numbers in offspring fed HE diet. The increase of adipose tissue CD206+CD11c+ ATMs in offspring fed HE diet was reversed by consuming a LP diet. Similar to HE treatment, LP increased *IL-6* expression in ATMs of offspring rats fed NE diets. However, this effect was diminished when offspring was fed on HE diet, which suggested an interaction between prenatal LP and postnatal HE diets on *IL-6* and *IL-1β* transcription.

Compared to a severe 4% protein [36], maternal 8% protein undernutrition diet as used in our current study do not cause significant decrease in key fetal growth factor hormone concentration alterations [36]. In addition, as shown in other studies, rat offspring exposed to 8% prenatal modest protein restricted diet do not have abnormal kidney functions or blood pressure while a more severe 5% prenatal protein restriction caused offspring kidney dysfunction and hypertension [37–41]. Therefore it is plausible 5% prenatal protein diet restriction would significantly increase the numbers of CD68+ or CD206+ cells compared to NP group adipose tissue. However, since the severe protein restriction leads to multi-organ dysfunction, the changes in plasticity of macrophages, if observed, will be more due to a compensatory or secondary effect rather than directly driven by maternal protein restriction. Thus, we choose to use a moderate protein restriction to study how prenatal and postnatal diets influence the ATM phenotype.

According to the “phenotypic switch” model of ATM, obesity leads to an accumulation of macrophages in the adipose tissue with M1-dominant phenotype, while ATM in non-obese subjects possess the M2-dominant phenotype. This is caused by direct activation of newly attracted macrophages/monocytes, rather than a phenotypic switch among resident macrophages [27, 42]. In our study, infiltration of ATM was not affected by the LP diet as evidenced by the lack of difference in the number of CD68^+^ cells in adipose tissue. However, upon postnatal HE treatment, there were less M1b subtype ATMs (CD11c+CD206+) in F1 offspring from the dam of maternal LP comparing to those from the maternal NP diet. Therefore, maternal LP diet interacts with postnatal HE diet to impact on the existing ATM phenotype, although prenatal LP diet may not influence the migration ability of adipose tissue monocyte/macrophages in F1 offspring of rats.

The increased expression of *IL-6* in ATM of LP offspring suggested that prenatal LP was capable of inducing macrophage inflammation. Consistently, two-way ANOVA also indicated increased expression of *IL-1β* by maternal LP diet. Interestingly, this effect was diminished under the condition of postnatal HE, which is consistent with previous report that the expression profile of genes associated with inflammation was reduced in the visceral adipose tissue of offspring rats from a dam with maternal protein restriction [43]. These data suggested a potentially antagonistic effect of maternal protein restriction and postnatal high energy on *IL-6* and *IL-1β* expression.

However, these interesting observations seemed to suggest that the prenatal LP diet is differentially affecting ATM phenotype with or without postnatal diet. To be noted, our data demonstrated a smaller adipocyte size in LP offspring and a positive correlation between adipocyte size and M1b/M1 ratio. A relationship between adipose tissue macrophage accumulation and adipocyte size has been demonstrated in many adipose tissue depots [44]. When fat cells reach a critical size, signals are generated to promote the release of pro-inflammatory cytokines and free fatty acids (FFA) resulting in recruitment of macrophages and a switch in macrophage phenotype [32, 44] Therefore, an increase in adipocyte size might be a necessary condition for adipocytes to synthesize and release proteins that regulate ATM heterogeneity. In our study, prenatal LP significantly reduced the wean body weight and wean fat however leads to catchup growth of the body weight and body fat mass, implying a size catchup of the LP adipocytes with persistent postnatal HE diet.

The feed efficiency (body weight gain/ energy intake) is an important contributing factor in adipose tissue weight gain. Thus, we compared feed efficiency across all 4 experimental groups. Our calculation showed (data not shown) that food efficiency do not explain additional increase in adipose tissue increase in LPHF (and LPNF to a lesser extent) group suggesting possible epigenetic maternal influence in adipose tissue growth.

**Fig 6:**
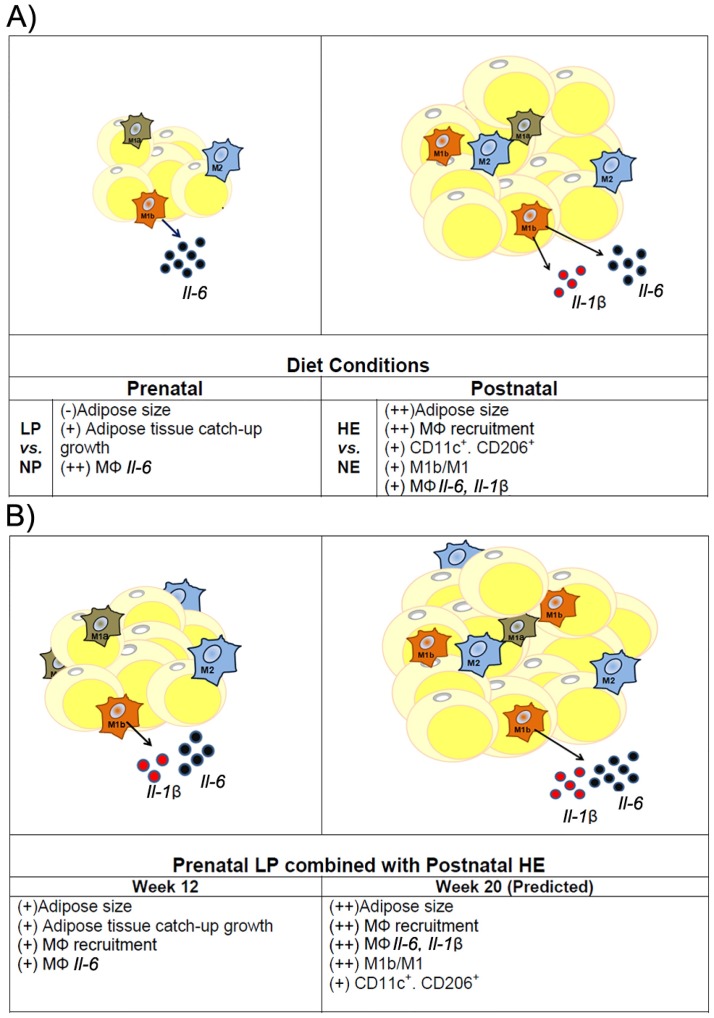
Model of LP and HE effects on macrophage activation and plasticity. A) Prenatal LP decreases the size of adipocytes, while increases adipose tissue catch-up growth. LP by itself also inhibited *IL-6* expression in ATMs, which is independent of the adipocyte size. Postnatal HE increases the size of adipocytes, which causes the recruitment of M1, especially m1b macrophages to the adipose tissue. These ATMs expressed more *IL-6* and *IL-1β*. B) When prenatal LP diets are combined with postnatal HE diets for 12 weeks, adipocyte is enlarged but is still smaller than NP+HE rats. There is induced adipose tissue inflammation in LP+HE rats; however, they have less ATM infiltration and decreased *IL-6* in ATMs comparing to that of NP+HE rats, which were correlated with smaller size of adipocytes. Considering that LP+HE rats have largest adipose tissue-catch up growth, it is predictable that prolonged HE diets on LP offspring for 20 weeks will eventually break up the temporary balance due to significantly enlarged adipocytes.

We therefore describe our model as follows (Fig 6). In juvenile LP+HE fed rats, despite fast growth of adipose tissue, there is a relatively lower grade of chronic inflammation in this adipose tissue than the juvenile NP+ HE offspring. This is correlated with the smaller adipocyte size, lower numbers of CLS in adipose tissue, and M1b subtype ATMs that are the major M1 subset contributing to the secretion of pro-inflammatory cytokines and insulin resistance [32]. Because prenatal LP leads to catchup growth of the body weight and adiposity[45], which might be associated with increased *Igf2* expression in LP offspring [46], it can be reasonably predicted that persistent HE diets from the juvenile stage to adult stage in LP offspring will eventually exceed the maximum capacity of adipose tissue to adapt to excess energy and lead to severe insulin resistance. The increased expression of *IL-6* in ATMs caused by LP diets will exacerbate insulin resistance in adult rats fed HE diets. This model is in consistent with previously reported studies that alterations in insulin resistance in response to LP prenatal and HE postnatal diets before and after 5 months of age [46, 47]. Extended treatment of postnatal HE diets on the same model of prenatal protein restriction in future studies will help to support the validity of this model.

In conclusion, we provide evidence that prenatal protein restriction preprogram the ATMs by increasing expression of the pro-inflammatory genes *IL-6* and *IL-1β* in ATMs. The prenatal protein restriction plays a synergic role with the postnatal high energy diet on the catch up growth and the enlargement of the adipocyte, which further correlates with the ATM plasticity and adipose tissue inflammation.

## Abbreviations

ANOVA: analysis of variance
ATM: macrophages
CLS: crown like structure
H&E: Haematoxylin Eosin
HE: high fat/energy diet (45% fat)
IUGR: induced intrauterine growth restriction
LBW: low birth weight
LP: low protein (8%)
NE: normal fat/energy diet (20% fat)
NP: normal protein (20% protein)
PBS: phosphate-buffered saline
SEM: standard error of the mean
SVC: Stromal Vascular Cell
